# Hypermethylation of *EDNRB* promoter contributes to the risk of colorectal cancer

**DOI:** 10.1186/1746-1596-8-199

**Published:** 2013-12-10

**Authors:** Cheng Chen, Lingyan Wang, Qi Liao, Yi Huang, Huadan Ye, Fei Chen, Leiting Xu, Meng Ye, Shiwei Duan

**Affiliations:** 1 Zhejiang Provincial Key Laboratory of Pathophysiology, School of Medicine, Ningbo University, Ningbo, Zhejiang 315211, China; 2 The Affiliated Hospital, Ningbo University, Ningbo, Zhejiang 315000, China; 3 Bank of Blood Products, Ningbo No.2 Hospital, Ningbo, Zhejiang 315010, China; 4 Department of Neurosurgery, Ningbo First Hospital, Ningbo University, Ningbo, Zhejiang 315010, China

**Keywords:** Colorectal cancer, *EDNRB*, DNA methylation, Biomarker

## Abstract

**Objective:**

Colorectal cancer (CRC) is one of the most common digestive malignancies in the world. *EDNRB* is a new candidate tumor suppressor gene which is often down-regulated or even silenced by promoter hypermethylation in various human cancers. However, the function of *EDNRB* gene in CRC remains unknown. In this study, we examined the expression and DNA methylation of *EDNRB* in CRC tissues.

**Methods:**

A total of 42 paired CRC and adjacent normal tissue samples were used to determine mRNA levels and DNA methylation status of *EDNRB* gene by qRT-PCR and methylation-specific PCR (MSP), respectively.

**Results:**

Our study showed that *EDNRB* promoter hypermethylation was more frequently in CRC tissues than in the normal tissues (92.86 versus 59.52, p = 0.001). Consequently, significantly lower level of *EDNRB* mRNA was found in CRC tumor samples than in normal samples (0.31 ± 0.91 versus 0.70 ± 1.18, p = 0.032).

**Conclusions:**

Our results suggested that *EDNRB* promoter hypermethylation might downregulate its gene expression in CRC, and thus played an important role in the development of CRC.

**The virtual slide:**

The virtual slides for this article can be found here: http://www.diagnosticpathology.diagnomx.eu/vs/7420980471113303

## Introduction

Colorectal cancer (CRC) is a malignant disease caused by a variety of factors involving with the accumulation of genetic and epigenetic changes. The incidence of CRC is increasing rapidly, and there are 1 million new CRC cases annually [1]. In the developed countries, the mortality of CRC has soared up to 33. CRC has become the second most common cancer in the world [2], accounting for 9 of all malignant tumors deaths [3]. Although 5-year survival rate of CRC patients has been improved from 22 to 47 in the last 30 years due to the advancement of the early diagnosis, surgical techniques and adjuvant therapies, the overall survival rate remains disappointing.

Abberrant expression of some important genes, such as *beta-catenin*[4], *SATB1*[5] and *EGFR*[6], were shown to be significantly associated with the occurrence and prognosis of CRC. Gene promoter hypermethylation often silences gene expression, while promoter hypomethylation tends to activate gene transcription. DNA methylation alteration has been considered as an important event in many malignancies including CRC.

Endothelin receptor type B (*EDNRB*) gene is located on 13q22, encoding a nonselective endothelin B receptor (ET_B_R) which belongs to a super-family of G-protein coupled receptor that mediates endothelins (ETs) [7]. ET_B_R is able to promote the production of neural crest cell-specific lineage, and thus it is related to the occurrence of Hirschsprung’s disease [8] that is a blockage in the colon.

There are massive CpG dinucleotide repeats in the 5′-flanking region of *EDNRB* gene. The methylation of this CpG-riched region was shown to be able to regulate *EDNRB* gene expression [9-11]. Hypermethylation of *EDNRB* gene promoter has been observed in leukemia [12], oral cancer [13], skin cancer [14], head and neck cancer [15], melanoma [16], renal cell carcinoma [17], bladder cancer [18] and prostate cancer [19]. However, the function of *EDNRB* gene in CRC remains unknown. In light of previous findings in various cancers, we investigate whether *EDNRB* gene promoter methylation contributes to the risk of CRC in Chinese population.

## Materials and methods

### Tissue samples collection

Tumor and its normal adjacent tissue samples were collected at the time of surgery from 42 primary sporadic CRC patients in the Department of Gastrointestinal Surgery in Affiliated Hospital of Ningbo University between June 2012 and April 2013. Normal adjacent tissues were collected at least 5 cm away from the tumor. Tissues were stored in liquid nitrogen at -80°C immediately after excision. The diagnoses of all CRC cases were pathologically confirmed. None of CRC patients had received preoperative chemotherapy or radiation therapy. Tumor stage was determined according to the Duke’s staging system, and cellular differentiation was graded according to the Broders’ grading system. All the patients in the study have signed the informed written consent forms.

### DNA isolation

Genomic DNA from tissue samples were extracted with QIAamp DNA Mini Kit (Qiagen, Hilden, Germany) according to the manufacturer’s instruction. Briefly, tissue sections were incubated with 180 μl of buffer ATL (in QIAamp DNA Mini Kit) and 20 μl of proteinase K on a thermostatic water bath at 56°C for 3 h, followed by incubation at 70°C for 10 min. Then, 200 μl of remixed Buffer AL (in QIAamp DNA Mini Kit) and ethanol (ratio 1:1) were added. Samples were mixed and transferred into QIAamp Mini spin columns. Centrifugation at 8,000 rpm for 1 min was followed by washing the spin column membrane with 500 μl of Buffer AW1 and 500 μl of Buffer AW2. DNA was eluted with 100 μl of Buffer AE. DNA concentration and quality were determined with spectrophotometer.

### Bisulfite modification

Eluted DNA was bisulphite-treated with ZYMO EZ DNA Methylation-Gold Kit according to the manufacturer’s instruction (Zymo Research, Orange, CA, USA). The bisulphite-modified DNA was resuspended in 10 μl of TE buffer.

### Methylation-specific PCR (MSP)

Methylation status of *EDNRB* promoter was determined by MSP. For the PCR reaction, 2 μl of modified DNA was amplified in a 20 μl reaction containing 0.3 μM each of forward and reverse primers, 0.2 mM dNTPs, 10 × PCR Buffer and 2.5 U of Hot Start DNA Polymerase (Qiagen, Hilden, Germany) under the following conditions: 15 min of denaturation at 95°C followed by 35 cycles of 45 s at 94°C, 45 s at 62°C for methylated primers, 1 min at 72°C and a final extension for 10 min at 72°C. The sequences of methylated and unmethylated primers were given in the previous study [20] (Table 1). Water blank was used as a negative control. PCR products were subjected to 2.0 agarose gel electrophoresis at 100 V for 20 min, and visualized directly under UV illumination. Samples were considered as methylation or unmethylation, when there were clearly visible bands (130 or 134 bp) on the gel for methylation or unmethylation primers.

**Table 1:** List of all primers used and conditions of PCR amplification

	**Primer**	**Sequence (5′-3′)**	**Product size (bp)**
M	F	CGAAGAGGTTGCGGGCGGTATTAGCG	130
	R	TACTCCAAAAACGTCCGATAACCG	
U	F	TGGTGAAGAGGTTGTGGGTGGTATTAGTG	134
	R	ACCTACTCCAAAAACATCCAATAACCA	
RT-PCR			
	EDNRB-F	GAAAGCCTCCGTGGGAATC	86
	EDNRB-R	ACAGCTCGATATCTGTCAATACTCAGA	
	GAPDH-F	CCATGGAGAAGGCTGGGG	194
	GAPDH-R	CAAAGTTGTCATGGATGACC	

### RNA isolation and reverse transcription

Total RNA was extracted from fresh frozen CRC and normal tissues from the same patients. RNA was extracted with TRIZOL reagent (Invitrogen Life Technologies Co, USA) according to the manufacturer’s protocol. RNA concentration and quality were determined by NanoDrop ND-1000 (Thermal Fisher Scientific, USA). The first-strand cDNA was synthesized according to the manufacturer’s instruction of M-MLV Reverse Transcriptase (Promega, Wisconsin, USA) with 2 μg DNase I-treated total RNA and 2 μM Oligo (dT)_15_ primer in a 20 μL volume.

### qRT-PCR

**Figure 1:**
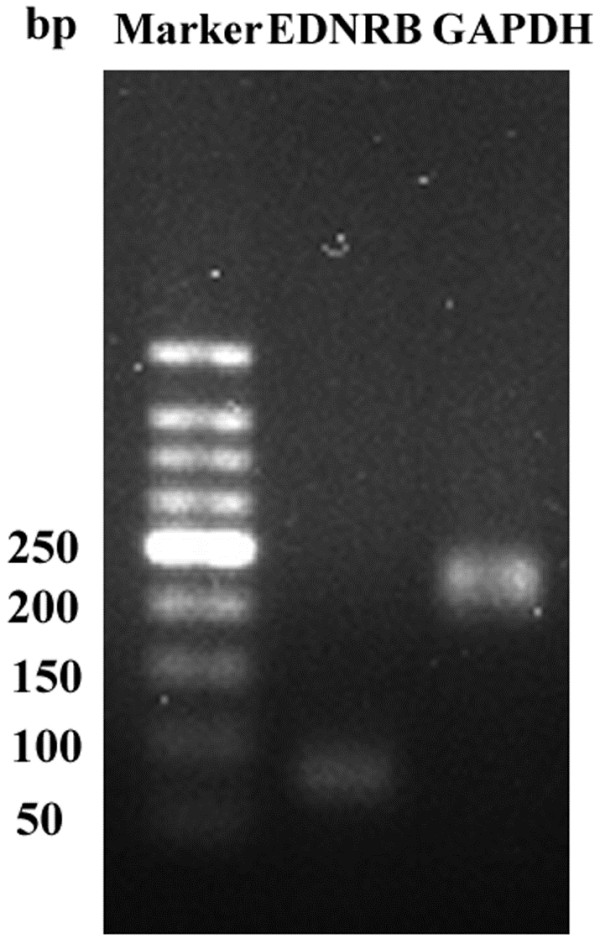
***EDNRB *** **and *GAPDH *** **mRNA expression in CRC tumor tissue.**

Primers for qRT-PCR were listed in Table 1[21]. The qRT-PCR reactions were conducted in a 96-well plate using ABI7500 Real-Time system (Applied Biosystems, CA, USA). Each reaction was performed in triplicate and in a 20 μL volume containing 2× real-time PCR Master Mix with SYBR Green dye (Promega, Wisconsin, USA), 0.4 μM of each primer and 4 μL cDNA, using the following thermal conditions: 95°C for 10 min, followed by 40 cycles of 95°C for 15 s, 60°C for 1 min and 72°C for 40 s. A melting curve analysis from 60°C to 95°C was performed at the end of each PCR to further confirm the specificity of amplicons. *GAPDH* was used as an endogenous control. The Ct values displayed by the instrument were recorded. Samples were confirmed whether there was a clearly visible band (86/194 bp) on the gel with *EDNRB* or *GAPDH* primers (Figure 1).

### Statistics

Statistical analysis was performed by the SPSS statistical package (version 16.0; SPSS, Chicago, IL, USA) and the results were presented using GraphPad prism software. Comparisons of *EDNRB* promoter methylation were performed by the correction formula of Chi-square test. The 2^–ΔCt^ method was used to analyze the result of qRT-PCR. Two groups of related data were analyzed using paired t-test. The Mann-Whiteney U test was used for two groups of independent data which did not meet normality test. All analyses were two-sided, and p < 0.05 was considered statistically significant.

## Results

### Hypermethylated *EDNRB* promoter in CRC

**Figure 2:**
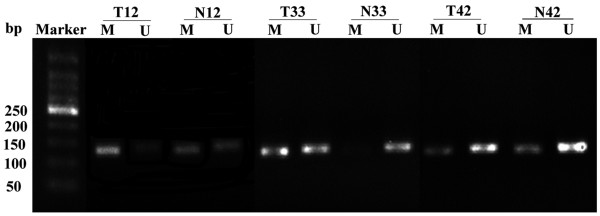
**Representative results for methylation status of *EDNRB *** **gene in CRC tumor tissues (T) and adjacent normal tissues (N).**

To determine methylation of *EDNRB* gene, MSP was performed on 42 CRC and 42 adjacent normal tissues in the primary sporadic CRC patients. The representative agarose gel electrophoresis results were shown in Figure 2. Methylation status was determined when there were methylated bands in the gel. And unmethylation status was determined if both the methylated and unmethylated bands were not detected, For the unmethylated samples, we repeated the relative experiment two times, including testing the DNA quality, bisulphite modification and MSP (confirming PCR primers quality and PCR conditions). If the methylated and unmethylated bands still did not detect, we confirmed the status of the sample as unmethylation. Our study showed that hypermethylation of *EDNRB* in CRC tissues was more frequently than in corresponding normal tissues (92.86 versus 59.52, p = 0.001, Table 2).

**Table 2:** **Methylation status of *EDNRB *** **gene in CRC and normal tissues**

	**Total**	**M**	**U**	**M%**	**χ**^**2**^	**p value**
CRC cases	42	39	3	92.86	11.091	0.001
Controls	42	25	17	59.52		

### Correlation of *EDNRB* methylation with clinicopathological characteristics

The relationship between methylation status of *EDNRB* gene and the clinicopathological characteristics of CRC was shown in Table 3. There was no significant difference in clinicopathological factors such as gender, age, TNM stages, lymph node status, metastasis status, tumor location, differentiation status, tumor size, and histological grade. There was no correlation of *EDNRB* gene methylation status with the serum levels of carcinoembryonic antigen (CEA) and carbohydrate antigen 19–9 (CA19-9).

**Table 3:** Association between the EDNRB methylation in CRC serum and clinicopathologicalfeatures

			**EDNRB Methylation**			
**Characteristics**		**n**	**M**	**U**	**χ**^**2**^	**p value**
Gender	Male	28	27	1	0.404	0.525
	Female	14	12	2		
Age(year)	≤60	16	16	0	0.629	0.428
	>60	26	23	3		
Stage	1/2	21	18	3	1.436	0.231
	3/4	21	21	0		
Lymph metastasis	Yes	21	21	0	1.436	0.231
	No	21	18	3		
Distant metastasis	Yes	8	8	0	0.012	0.913
	No	34	31	3		
CEA	≥5.0 ng/ml	15	14	1	<0.001	1
	<5.0 ng/ml	27	25	2		
CA19-9	≥37U/ml	9	8	1	<0.001	1
	<37U/ml	33	31	2		
Tumor location	Colon	26	24	2	<0.001	1
	Rectum	16	15	1		
Differentiation	Poor	10	10	0	0.091	0.763
	Moderate	32	29	3		
	Well	0	0	0		
Tumor size	<5 cm	28	25	3	0.404	0.525
	≥5 cm	14	14	0		
Histological classification	Adenocarcinoma	40	37	3	<0.001	1
	Mucinous adenocarcinoma	2	2	0		
	Undifferentiated carcinoma	0	0	0		

### *EDNRB* expression is down-regulated in CRC tissues

**Figure 3:**
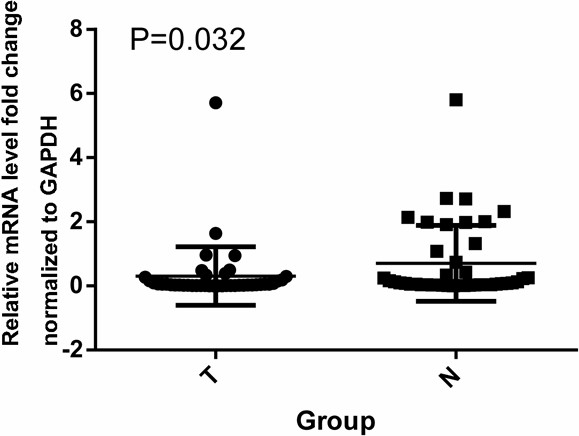
**Expression level of *EDNRB *gene in CRC tumor tissues (T) and adjacent normal tissues (N).** RT-qPCR was used to analyze the expression level of *EDNRB* gene in CRC tumor tissues and adjacent normal tissues. The relative expression of *EDNRB* mRNA level was significantly higher in normal tissues than that in CRC tumor tissues (p = 0.032).

We determined *EDNRB* mRNA expression in 42 CRC tissues and 42 adjacent normal colorectal tissues by qRT-PCR. The gene expression level of *EDNRB* was normalized with the values of the control gene *GAPDH*. Our results showed that *EDNRB* mRNA level in CRC tumor samples was significantly lower than in their adjacent normal samples (0.31 ± 0.91 versus 0.70 ± 1.18, p = 0.032, Figure 3, Table 4).

**Table 4:** ***EDNRB *** mRNA expression levels in CRC and normal tissues*

	**Total**	**Mean**	**SD**	**SE**	**t**	**p value**
CRC	42	0.3074	0.91	0.14	-2.214	0.032
Control	42	0.7041	1.18	0.18		

*: SD denotes standard deviation and SE denotes standard error.

### Correlation of *EDNRB* expression status with clinicopathological characteristics

We further examined the relationship between *EDNRB* mRNA expression and clinicopathological characteristics (Table 5). There was no significant correlation between *EDNRB* expression and the clinicopathological factors such as gender, age, TNM stages, lymph node status, metastasis status, tumor location, differentiation status, tumor size, and histological classification.

**Table 5:** **Correlation of *EDNRB *** mRNA expression and clinicopathological parameters of colorectal cancer samples*

			***EDNRB *** **expression relative to *GAPDH***			
**Characteristics**		**n**	**Mean**	**SD**	**SE**	**p value**
Gender	Male	28	0.336	1.073	0.202	0.722
	Female	14	0.250	0.480	0.130	
Age (year)	≤60	16	0.221	0.444	0.111	0.836
	>60	26	0.360	1.114	0.218	
Stage	1/2	21	0.143	0.233	0.051	0.792
	3/4	21	0.472	1.257	0.281	
Lymph metastasis	Yes	21	0.457	1.266	0.276	0.95
	No	21	0.158	0.238	0.052	
Distant metastasis	Yes	8	0.781	1.996	0.706	0.741
	No	34	0.196	0.351	0.060	
CEA	≥5.0 ng/ml	15	0.121	0.156	0.040	0.723
	<5.0 ng/ml	27	0.411	1.127	0.217	
CA19-9	≥37U/ml	9	0.102	0.153	0.051	0.526
	<37U/ml	33	0.364	1.023	0.178	
Tumor location	Colon	26	0.428	1.145	0.225	0.351
	Rectum	16	0.112	0.156	0.039	
Differentiation	Poor	10	0.719	1.779	0.558	0.494
	Moderate	32	0.179	0.341	0.062	
	Well	0	/	/	/	
Tumor size	<5 cm	28	0.386	1.102	0.208	0.968
	≥5 cm	14	0.151	0.248	0.066	
Histological classification	Adenocarcinoma	40	0.315	0.935	0.148	0.455
	Mucinous adenocarcinoma	2	0.162	0.157	0.111	
	Undifferentiated carcinoma	0	/	/	/	

*: SD denotes standard deviation and SE denotes standard error.

## Discussion

Cancer development and progression may be contributed by both genetic and epigenetic factors. As one of the major epigenetic modifications, DNA methylation of promoter often down-regulates gene transcription. A growing number of evidences show that DNA methylation mediated tumor suppressor gene silencing may contribute to tumor progression [22-24]. Aberrant DNA methylated loci have become promising biomarkers in the early diagnosis of diseases [25,26].

EDNRB is a G protein–coupled receptor that activates a phosphatidylinositol-calcium second messenger system. The product of *EDNRB* gene (ET_B_ receptor, ET_B_R) is able to bind to endothelins (ETs), consisting of a family of 3 potent vasoactive peptides (ET1, ET2, and ET3) [13]. During the development of tumor, *EDNRB* gene transcription is downregulated by promoter hypermethylated, and consequently alters the ET1 signaling pathway [27]. Disruption in the ET1 signaling pathway has been shown to be involved in a variety of human tumor proliferation, angiogenesis, and metastasis [28-30]. *EDNRB* gene silencing by promoter hypermethylation has been reported in a variety of tumors such as leukemia, oral cancer, skin cancer, head and neck cancer, melanoma, renal cell carcinoma, bladder cancer, and prostate cancer [12-19]. Our study in CRC adds a new piece of evidence for the contribution of *EDNRB* promoter hypermethylation to the risk of CRC. Our results further confirmed that DNA methylation in the promoter region played a key role in *EDNRB* transcription.

Some reports have focused on the correlation between *EDNRB* methylation and cancer clinical features [31-33]. Hypermethylation of the *EDNRB* gene in paired gastric cancer tissues and adjacent normal tissues from 96 patients was detected [32]. *EDNRB* gene promoter methylation was shown to be associated with gastric cancer tumor invasion [32]. The extent of hypermethylation at CpG island in *EDNRB* gene was also evaluated in seven prostate cancer cell lines, normal prostate epithelial cells, normal prostate stromal cells, 73 primary prostate cancers, 91 metastatic prostate cancers, and 25 noncancerous prostate tissues [33]. *EDNRB* CpG island hypermethylation was shown to be correlated with pathological stage and Gleason score to a statistically significant extent in prostate cancer [33]. In the present study, we examined the relationship between the *EDNRB* methylation and the clinical features. Unfortunately, there was no significant relationship between *EDNRB* methylation or expression status and the clinical features. And this may be due to a lack of power in our samples. As a kind of glycoprotein produced by colorectal cancer tissue, CEA is a good tumor marker for judging efficacy, disease progression, and prognosis of colorectal cancer. CA19-9 was frequently increased in gastrointestinal tumors such as pancreatic cancer, gastric cancer, and colorectal cancer. CEA and CA19-9 are commonly and traditionally used in clinical colorectal cancer detection. Our study showed no correlation of *EDNRB* gene methylation status with the serum levels of CEA and CA19-9. This might imply that aberrant *EDNRB* methylation and conventional tumor markers could serve as complementary markers in the CRC diagnosis, although further work is needed to confirm our hypothesis.

In conclusion, we revealed a significant contribution of *EDNRB* hypermethylation to the risk of CRC. These findings may provide a new clue for detection and treatment of CRC. Future research is needed to determine the detailed mechanism of *EDNRB* gene in the risk of CRC.

## Abbreviations

CRC: Colorectal cancer; EDNRB: Endothelin receptor type B; ETAR: Endothelin A receptor; ETBR: Endothelin B receptor; ETs: Endothelins.

## Competing interest

None of the authors have any commercial or other associations that might pose a conflict of interest. All authors are responsible for the content and writing of the paper.

## Authors’ contributions

MY and SD participated in research design. YH, HY and FC conducted experiments. LW, QL and LX performed data analysis. The manuscript was drafted by CC and SD, and critically reviewed and discussed with the other co-authors. All the authors read and approved the final manuscript.

## Authors’ information

Cheng Chen, Lingyan Wang, Qi Liao: co-first authors of this work.
